# 900. The Epidemiology of Viral Coinfections within Households with Children

**DOI:** 10.1093/ofid/ofad500.945

**Published:** 2023-11-27

**Authors:** Jessica C Ibiebele, Emily T Martin, Arnold Monto, Amy Callear

**Affiliations:** University of Michigan School of Public Health, Canton, Michigan; University of Michigan, Ann Arbor, MI; University of Michigan, Ann Arbor, MI; University of Michigan School of Public Health, Canton, Michigan

## Abstract

**Background:**

Improvements in virus detection using molecular assays, such as multiplex polymerase chain reaction, has led to more common identification of viral coinfections. The incidence of viral coinfections may depend on population factors, such as age structure, distribution of chronic respiratory conditions, geographic location, and setting of the study population (e.g., community, in-patient admission), which has new relevance given observations of coinfections involving SARS-CoV-2. Household-level data on mild to moderate illnesses offer an important complement to other coinfection data that are largely measured in clinical settings.

**Methods:**

The Household Influenza Vaccine Evaluation (HIVE) study is an ongoing prospective cohort study of households with children in Southeast Michigan that began in 2010. Active acute respiratory illness (ARI) surveillance is conducted, and participants with eligible ARI’s are tested for a range of respiratory pathogens. Descriptive statistics were used to characterize viral coinfections within this cohort for a period prior to the COVID-19 pandemic (2010-2020) and will be performed for a period during the pandemic (2020-2022). The consequences of these coinfections in terms of symptoms and household transmission are under evaluation.

**Results:**

There were 6,245 virus-positive ARI’s in the cohort from 2010-2020, with multiple viruses detected in 896 (14.3%) ARI’s. Of the coinfections, rhinovirus/enterovirus most frequently appeared (58.15%), followed by common CoV (38.84%), and AdV (24%) (See Figure 1). The most common virus combination was a coronavirus (229E, OC43, NL63, or HKU1) along with rhinovirus/enterovirus, making up 12.05% (n=108) of coinfections. Eight infections were made up of four or more viruses. Similar analyses will be conducted for pandemic-period data.

**Figure:**
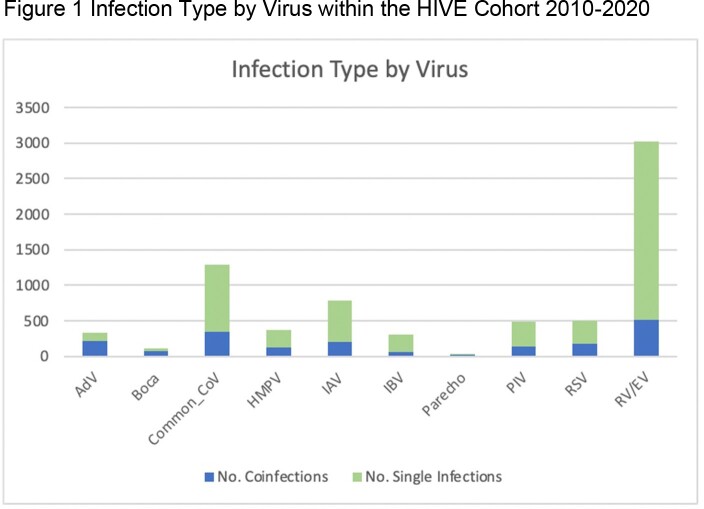

**Conclusion:**

While awareness of viral coinfections has grown since the start of the COVID-19 pandemic, coinfections within the HIVE cohort were not uncommon prior to the pandemic. Coronaviruses were the second leading viruses identified in coinfections, which aligns with observations noted during the pandemic. These results contribute important information about coinfections and expand the understanding of respiratory virus dynamics within households.

**Disclosures:**

**Emily T. Martin, PhD, MPH**, Merck: Grant/Research Support **Arnold Monto, MD**, Roche: Advisor/Consultant|Roche: Honoraria

